# Hyepereosiniphilic syndrome and COVID-19: 2 case reports

**DOI:** 10.1186/s13019-023-02241-1

**Published:** 2023-04-21

**Authors:** Alborz Sherafati, Mehrzad Rahmanian, Roya Sattarzadeh Badkoubeh, Meysam Khoshavi, Morteza Foroumandi, Soheil Peiman, Farhad Shahi, Akram Sardari, Roghayeh Pourkia, Farnoosh Larti

**Affiliations:** 1 Cardiology Department, Imam Khomeini Hospital, Tehran University of Medical Sciences, Keshavarz Boulevard, P.O. Box: 1419733141, Tehran, Iran; 2 Department of Cardiovascular Surgery, Imam Khomeini Hospital, Tehran University of Medical Sciences, Tehran, Iran; 3 Anesthesiology and Intensive Care Department, Imam Khomeini Hospital, Tehran University of Medical Sciences, Tehran, Iran; 4 AdventHealth Orlando Hospital, Orlando, FL USA; 5 Department of Hematology and Medical Oncology, Tehran University of Medical Sciences, Tehran, Iran; 6 Department of Cardiology, School of Medicine, Rouhani Hospital, Babol University of Medical Sciences, Babol, Iran

**Keywords:** Hypereosinophilic syndrome, COVID-19, Prosthetic valve thrombosis, Cardiogenic shock

## Abstract

**Background:**

Nearly half of the patients with hypereosinophilic syndrome (HES) have cardiovascular involvement, a major cause of mortality. COVID-19 infection can lead to cardiac involvement, negatively impacting the clinical course and prognosis. We reported two patients with HES complicated by COVID-19, with cardiac involvement and valve replacement.

**Case presentation:**

Our first patient was a 27-year-old woman admitted due to dyspnea and signs of heart failure. She had severe mitral stenosis and mitral regurgitation on the echocardiogram. Corticosteroid therapy improved her symptoms initially, but she deteriorated following a positive COVID-19 test. A repeated echocardiogram showed right ventricular failure, severe mitral regurgitation, and torrential tricuspid regurgitation and, she underwent mitral and tricuspid valve replacement. Our second patient was a 43-year-old man with HES resulted in severe tricuspid stenosis, which was improved with corticosteroid treatment. He underwent tricuspid valve replacement due to severe valvular regurgitation. He was admitted again following tricuspid prosthetic mechanical valve thrombosis. Initial workups revealed lung involvement in favor of COVID-19 infection, and his PCR test was positive.

**Conclusion:**

COVID-19 infection can change the clinical course of HES. It may result in a heart failure exacerbation due to myocardial injury and an increased risk of thrombosis in prosthetic valves or native vessels due to hypercoagulability.

**Supplementary Information:**

The online version contains supplementary material available at 10.1186/s13019-023-02241-1.

## Background

Hypereosinophilic syndrome (HES) is defined as a high peripheral eosinophil count (> 1.5 × 10^9^/L), along with evidence of eosinophil-related organ damage [1]. The etiology can be neoplastic (primary), reactive to conditions like parasitic infection or lymphoma (secondary), and, more commonly, idiopathic [1]. Cardiac involvement, known as “Loeffler’s Endocarditis,“ consists of three phases: acute necrotic asymptomatic phase, thrombotic phase with possible embolic events, and fibrotic phase with restrictive cardiomyopathy and symptoms of heart failure [1, 2]. Nearly half of the patients have cardiovascular involvement, a major cause of mortality in HES [3].

Over the past two years, millions have been affected by coronavirus disease 2019 (COVID-19). Although upper respiratory tract symptoms are the most common manifestation, myocardial injury can occur in many patients, ranging from myocarditis to acute heart failure and cardiogenic shock [4]. Cardiac involvement negatively affects the clinical course and prognosis of COVID-19, especially in patients with pre-existing heart disease. COVID-19 also exacerbates the compensated pre-existing cardiac diseases or increases complications [4]. Herein, we describe two patients with HES complicated by COVID-19 and discuss the coagulation status and treatment strategies in such patients.

## Case presentation

### Case 1

A 27-year-old woman was admitted to our hospital for dyspnea at rest and fatigue. Her symptoms had increased gradually during the few weeks before admission. She also had nausea, poor appetite, and palpitation. In her past medical history, a history of controlled asthma with inhaled corticosteroids was present for ten years. Her habitual history was unremarkable. In physical examination, her blood pressure was 100/60 mmHg, heart rate was 103 beats per minute, respiratory rate was 30 per minute, oral temperature was 36.7 C, and oxygen saturation was 96% in room air. Jugular venous pressure (JVP) was elevated, and a systolic murmur was heard in the lower left sternal border and cardiac apex. Breath sounds were reduced in the basal parts of the lungs. Bilateral lower limb pitting edema was detected in both legs.

In the initial lab data, complete blood count (CBC) with differential was as follows: hemoglobin (Hb) level was 12.7 g/dl, platelet count was 109,000 × 10^9^/L, and white blood cell (WBC) count was 13,500 × 10^9^/L mm3 with the Neutrophils and lymphocytes percentages equal to 49% and 12%, respectively. The eosinophil count was significantly high (4185 × 10^9^/L, 31% of WBCs). Chest X-ray showed bilateral pleural effusion. Pleural fluid analysis showed a protein level of 1200 mg/dl, a lactate dehydrogenase (LDH) level of 297 U/L, and an albumin level of 800 mg/dl. Simultaneous blood sampling showed a serum LDH level of 558 U/L, a serum total protein level of 6.4 g/dl, and a serum albumin level of 3.6 g/dl. The results were in favor of a transudate effusion. Spirometry was performed based on the history of asthma, which showed a forced expiratory volume (FEV1) of 37%, a forced vital capacity of 39%, and an FEV1 to FVC ratio of 92%. The pattern was in favor of mixed obstructive and restrictive airway disease.

**Fig. 1:**
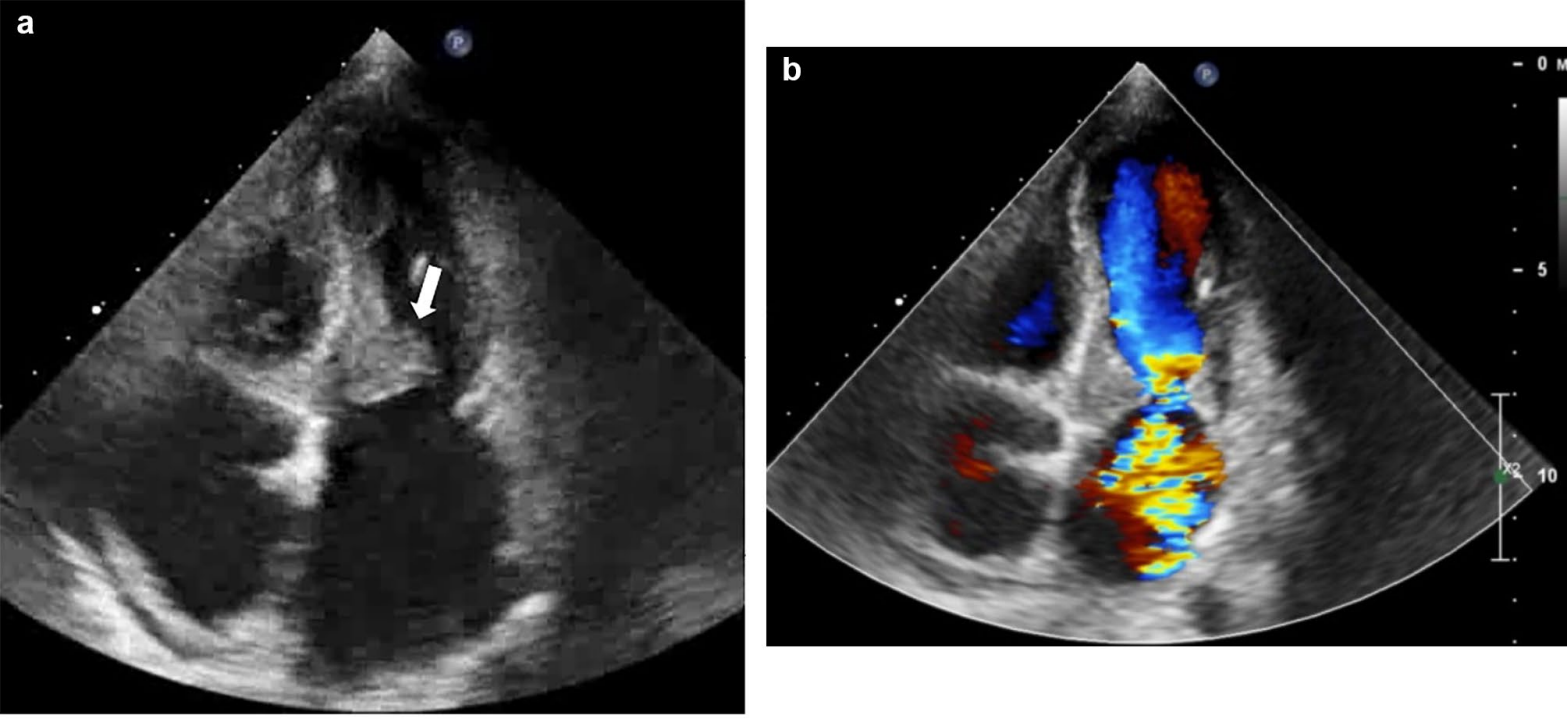
Apical 4-chamber view. An increased thickness in the ventricular side of mitral valve leaflets is seen (A, arrow), resulting in mass formation. In addition, severe mitral regurgitation is also present (B)

Cardiac auscultation findings and a transudate pleural effusion highlighted a cardiac etiology for her complaints. Echocardiography was performed in the next step, which showed a left ventricular ejection fraction (LVEF) of 55%. There was a significantly increased thickness in the ventricular side of mitral valve leaflets that was extended to the basal part of the ventricular endocardium, resulting in mass formation (Video S1). The maximum diameter was about 20 mm (Fig. 1). Severe functional mitral stenosis (MS) with a mean gradient of 16 mmHg and severe mitral regurgitation (MR) were detected (Video S2). Slight obliteration of RV apex was evident with sparing of LV apex. Tricuspid valve leaflets were thickened and mal-coapted with severe tricuspid regurgitation (TR) and no tricuspid stenosis. The inferior vena cava (IVC) was dilated with reduced respiratory collapse. Systolic pulmonary arterial pressure (PAP) was 43 mmHg. The patient was diagnosed with HES regarding high eosinophil count and echocardiographic findings.

Extensive diagnostic workups were performed to elucidate the etiology of high eosinophil count, including peripheral blood smear (PBS) preparation and additional blood tests. PBS was unremarkable except for an elevated eosinophil count. Vitamin B12 level was 510 pg/mL (200–835 pg/mL). Other lab data were as follows: Creatinine level was 1.1 mg/dl, Aspartate aminotransferase (AST) level was 17 U/L, and alanine aminotransferase level was 25 U/L, Anti-nuclear antibody (ANA) level was 0.2 (< 1.0), C-anti neutrophilic cytoplasmic antibody (ANCA) level was 0.6 U/mL (< 18), P-ANCA level was 0.5 U/mL (< 18), and interferon-gamma release assay (IGRA) for tuberculosis was negative. Urine analysis was normal. Abdominal sonography showed normal liver and spleen size and echogenicity with mild to moderate ascites. Stool examination showed no parasite ova or larva. No evidence of malignancy or primary myeloproliferative disorder was detected in bone marrow aspiration and biopsy. Evaluation for genetic translocation of BCR/ABL and ETV6-PDGFRB were also negative. The results were in favor of idiopathic HES. Results of the cardiac biomarkers were as follow: N terminal pro B type natriuretic peptide (NT-pro BNP) level was 46 pg/ml, and high sensitive troponin I (hs-CTnI) level was 0.015 mcg/L (< 0.12).

Corticosteroid therapy and intravenous heparin were initiated, with a relative improvement in symptoms after one week. WBC count and eosinophil percentage were decreased to 8000 × 10^9^/L and 0.3%, respectively. However, clinical deterioration occurred during the hospital course and after initial improvement. The oxygen saturation was reduced to 80%. Chest CT scan showed ground glass opacification in favor of COVID-19. Nasopharyngeal swab polymerase chain reaction (PCR) was positive for SARS-COV-2. the erythrocyte sedimentation rate (ESR) was increased to 63 mm/h, and the D-dimer level was 830 mcg/L. Supportive medical therapy was initiated and continued for a week. However, systolic blood pressure was decreased to 80 mmHg with evidence of end-organ hypoperfusion that mandated the administration of an intravenous inotropic agent (norepinephrine). Repeated bedside echocardiography showed significant right ventricular (RV) dysfunction with torrential TR and severe MR. As severe MR and torrential TR both played key roles in the clinical picture of the biventricular failure, the patient underwent cardiac surgery with bioprosthetic tricuspid and mitral valve replacement. After the surgery, she had an uneventful hospital stay and was discharged home after one week.

### Case 2

**Fig. 2:**
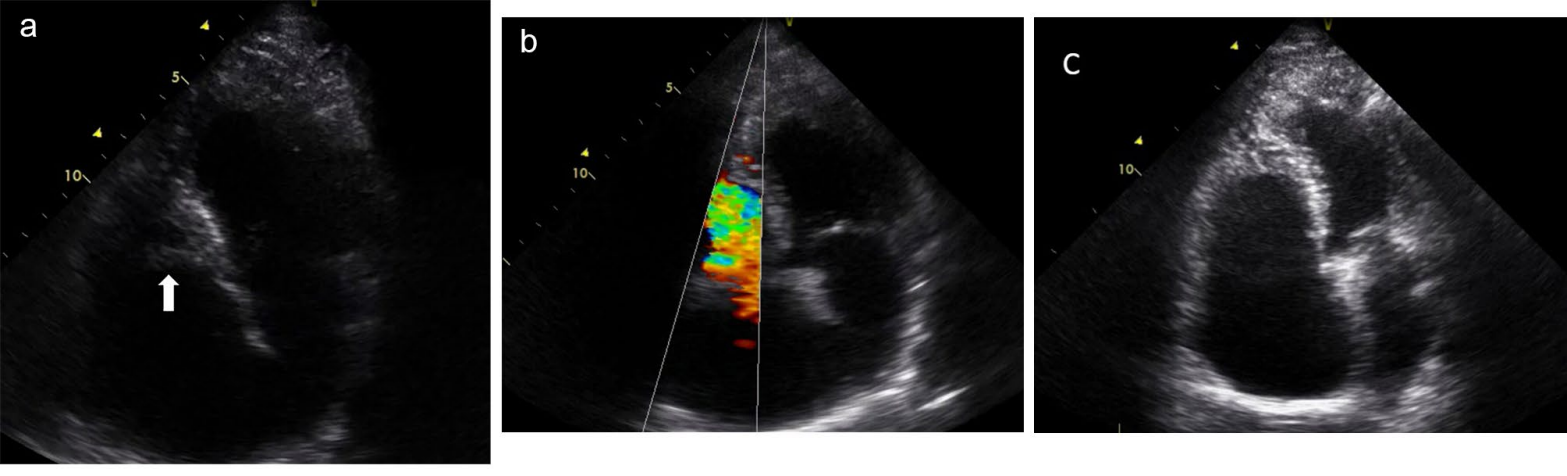
Apical 4-chamber view. Severe thickening of tricuspid valves with extension to the RV free wall is seen (A, arrow), resulting in an increased gradient and severe tricuspid stenosis (B). However, a follow-up echocardiogram two months later shows no evidence of tricuspid valve thickening and stenosis (C)

A 43-year-old man was admitted to our hospital in 2014 due to severe dyspnea and cough initiated one week before admission. He had New York Heart Association (NYHA) class III exertional symptoms. However, there was no history of fever or other constitutional symptoms. The patient had been treated for asthma and chronic sinusitis for over a decade. In physical examination, ascites and bilateral peripheral pitting edema were detected. Paracentesis showed high serum ascites albumin gradient (SAAG). WBC count was 22,000 × 109/L, with a high eosinophil count (11,000 × 109/L). Echocardiography showed an LVEF of 40%, prominent obliteration of RV apex and, to a lesser degree, LV apex, as well as severe thickening of tricuspid valves with extension to the RV free wall resulting in severe tricuspid stenosis (TS) with a mean gradient of about 7 mmHg, and mild to moderate TR (Fig. 2, Videos S3–S5). Extensive thickening of pulmonary leaflets was also evident, with no pulmonary hypertension. The IVC was plethoric.

The patient was diagnosed with HES, and secondary causes of eosinophilia, including parasitic infections, myeloproliferative disorders, and rheumatologic diseases, were ruled out. Results of BCR/ABL translocation and ETV6-PDGFRB were negative. Corticosteroid therapy was initiated, resulting in eosinophil count decline and improvement in symptoms. Two months later, the follow-up echocardiogram revealed a severe TR with no evidence of TS (Fig. 2C), and the patient underwent tricuspid valve replacement with a mechanical bileaflet prosthesis. The decision to place a mechanical valve was based on the patient’s preference despite the higher probability of valve thrombosis due to his underlying disease. He had been on warfarin 5 mg daily since then, and the international normalization ratio (INR) was in the therapeutic range (mean INR ~ 3). He had two episodes of mechanical valve thrombosis with minimal symptoms in the following 6 years that were detected in the routine follow-up echocardiograms and were treated with intravenous thrombolysis (both episodes occurred in the course of bridge therapy for non-cardiac surgery and a dental procedure).

In September 2020, the patient was admitted with severe dyspnea and peripheral edema. On physical examination, his blood pressure was 110/80 mmHg, heart rate was 92 bpm, respiratory rate was 30/min, and oral temperature was 37.8 C. Jugular venous pressure was elevated. Prosthetic valve sound was not heard clearly, and scattered crackles were heard during lung auscultation. Other examination findings were unremarkable. In the lab data, the WBC count was 11,830 × 109/L, the Hb level was 13.7 g/dl, and the platelet count was 185,000 × 109/L. The eosinophil count was high (23%). Echocardiography showed an LVEF of 40%, fixed tricuspid prosthesis leaflets, and a significant RV dysfunction. Fluoroscopy was performed, which confirmed the fixation of both tricuspid prosthesis leaflets (Video S6, S7). The INR was 1.84 (the previous documented INR one month before admission was 3; the patient did not change his warfarin dose, did not start a new medication, or did not change his diet since then). Cardiac biomarkers were as follows: NT-pro BNP was 3832 pg/mL, and hs-CTnI was 0.096 mcg/L. Other lab data were as follows: creatinine level was 1.4 mg/d, ANA was 0.2, C- ANCA was 0.9 U/mL, P-ANCA was 12.5 U/mL, and sputum smear was negative for tuberculosis. Abdominal sonography showed normal liver and spleen size and echogenicity.

**Fig. 3:**
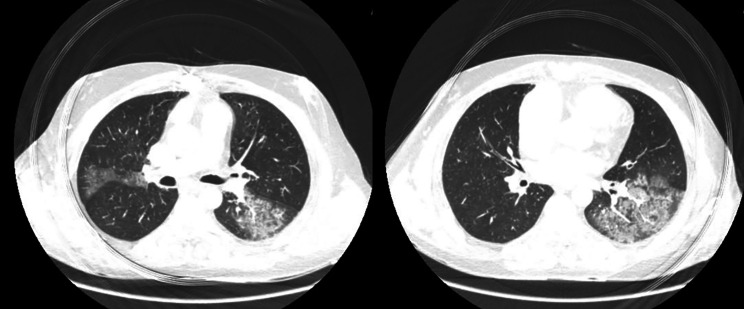
Chest CT scan shows ground glass opacities mostly in left lung in favor of COVID-19 infection

There was a high suspicion of pulmonary embolism from tricuspid valve thrombosis as the etiology of the patient`s dyspnea. COVID-19 was in the differential diagnosis. Chest CT was ordered, revealing evidence of segmental pulmonary embolism along with ground glass appearance and consolidation, suggesting COVID-19 (Fig. 3). PCR for SARS-COV-2 was requested, which was positive. Corticosteroid therapy was initiated due to a high eosinophil count. As the risk of redo cardiac surgery was unacceptable, an infusion of low-dose alteplase (25 mg in 25 h) was administrated for tricuspid valve thrombosis. The fibrinolytic therapy was stopped due to hemoptysis in the early hours of alteplase infusion. Therapy with corticosteroids was continued with no further intervention due to the patient`s normal oxygen saturation and stable hemodynamics. The eosinophil count declined, and the patient underwent an elective redo bioprosthetic tricuspid valve replacement following a negative PCR test. The post-operation course was uncomplicated, and the patient was discharged one week after surgery.

## Discussion

Choosing a treatment strategy for HES depends on the underlying etiology. This necessitates a full diagnostic workup before initiating any treatments. If no specific underlying condition is found in the diagnostic tests, as in our patients, a corticosteroid is the first line treatment of idiopathic HES [3, 5], resulting in the reduction of eosinophil counts and prevention of further organ damage [6]. Our patients, especially the second one, had a good initial response to corticosteroid therapy, and their eosinophil counts declined. Corticosteroid therapy resulted in regression of the TS in our second patient, a unique response not reported before. However, MR severity regression following corticosteroid therapy in HES was reported previously [7]. Although the valvular structures were damaged, and the patient eventually needed surgery due to the remaining TR, the surgery was elective following the resolution of symptoms and reduced eosinophil count. Corticosteroids were safe and effective in the admissions with concomitant COVID-19, resulting in a stable clinical course and improvement. The hemodynamic compromise in our first patient was possibly due to COVID-19 infection COVID-induced myocardial inflammation, resulting in further structural damage and valvular regurgitation intensification. Considering a trial of corticosteroids in HES patients with severe valvular involvement (including valvular stenosis) and stable hemodynamics before surgery is reasonable. However, surgical correction should be considered in the case of severe valvular involvement with significant hemodynamic decompensation despite initial medical treatment.

In patients with HES, high eosinophil count can decrease thrombomodulin level (by proteins secreted from their granules) and increase the expression of tissue factor (released from eosinophil granules) with consequent activation of factor VII and an increase in fibrinogen level [8, 9], leading to the activation of the coagulation cascade and hypercoagulability state [3]. The hypercoagulability state increases the risk of thrombosis in mechanical valves. Therefore, it is recommended to use bioprosthetic valves in these patients under 55–65 years despite the American College of Cardiology (ACC)/American Heart Association (AHA) guidelines [10] and the higher probability of valve degeneration and the need for future redo surgeries [1, 8]. It is also recommended to start warfarin with a therapeutic INR range after bioprosthesis implantation to reduce the risk of prosthetic valve thrombosis. Our first patient agreed to have bioprosthetic mitral and tricuspid valves, but our second patient didn`t accept the risk of redo surgery and had a mechanical tricuspid valve. However, he had three episodes of prosthetic valve thrombosis after the first surgery, and finally, a bioprosthetic valve was implanted.

Despite the hypercoagulability state, there is no specific recommendation on initiating prophylactic or therapeutic anticoagulation in HES with no valvular intervention [11]. There are case reports of portal venous thrombosis [11], deep venous thrombosis, and pulmonary embolism [12] in patients with HES. All these cases had high eosinophil counts, indicating an active disease status. These cases were treated with corticosteroids and anticoagulants with good clinical response. Our first patient was on complete bed rest, and we decided to initiate heparin infusion due to a concern for thrombosis formation following prolonged immobility, as well as hypercoagulability. Although low molecular weight heparin (LMWH) is another alternative [8], we decided not to use it due to its unknown anticoagulation effect in the HES.

COVID-19 results in myocarditis and myocardial damage in a subset of patients [13] due to the infiltration of immune cells and hyperactivation of the immune system with high cytokine release [4]. Pericardial involvement following a cytokine storm, resulting in cardiac symptoms, is also reported [14]. COVID-19 also alters the coagulation status. There are reports on increased D-dimer levels in COVID-19 patients, which is related to a worse prognosis [15, 16]. Fibrinogen level is also increased [15]. Increased pro-inflammatory cytokines, especially interleukin-6, can result in elevated tissue factor expression [15, 16]. Furthermore, angiotensin II pro-inflammatory activity can be dominant, including more tissue factor expression [16, 17]. Consequently, a hypercoagulability state and an increased risk of thrombotic complications occur. It is also possible that COVID-19 triggers HES flares, as was suggested in a case report of an acute exacerbation of idiopathic HES following asymptomatic COVID-19 [18].

Our second patient’s dyspnea was partly due to his lung infection and segmental pulmonary embolism. However, cardiac decompensation following the prosthetic valve thrombosis and pulmonary hypertension due to his lung involvement also played a significant role. Regarding his valvular thrombosis and pulmonary embolism, he was admitted with a high eosinophil count, reflecting an active disease status, and a concomitant COVID-19 disease, both associated with the hypercoagulability state, with possible additive effects. The patient`s INR was subtherapeutic on admission despite previous therapeutic INRs and no change in his surveillance, diet, or drugs. This decrease in INR could result from a factor VII enhancement activated by tissue factor since HES and COVID-19 are associated with higher tissue factor expression and activity.

## Conclusion

There are important considerations for the therapeutic management of HES, including the time of surgery, selection of the prosthetic valve, and prophylactic or therapeutic anticoagulation. Furthermore, we must be aware that the COVID-19 infection can change the clinical course of HES. For instance, it can exacerbate myocardial injury and heart failure, as in our first patient, or increase the risk of thrombosis in prosthetic valves or native vessels due to the hypercoagulability state. Therefore, closer surveillance of patients is needed in COVID-positive patients.

## Electronic supplementary material

Below is the link to the electronic supplementary material.

Video S1: Transthoracic echocardiography showed significantly increased thickness of the ventricular side of mitral valve leaflets with extension to the basal part of the ventricular endocardium; slight obliteration of RV apex with sparing of LV apex was also noted.

Video S2: Severe functional mitral stenosis with a mean gradient of 16 mmHg and severe mitral regurgitation was detected. Tricuspid valve leaflets were thickened and mal-coapted with severe tricuspid regurgitation and no evidence of tricuspid stenosis.

Video S3: Prominent obliteration of RV apex and, to a lesser degree, LV apex, as well as severe thickening of tricuspid valves with extension to the RV free wall resulting in severe tricuspid stenosis with a mean gradient of about 7 mmHg, and mild to moderate TR were observed.

Video S4: Prominent obliteration of RV apex and, to a lesser degree, LV apex, as well as severe thickening of tricuspid valves with extension to the RV free wall resulting in severe tricuspid stenosis with a mean gradient of about 7 mmHg, and mild to moderate TR were observed.

Video S5: Extensive thickening of pulmonary leaflets was evident, with no pulmonary hypertension.

Video S6: Fixation of both tricuspid prosthesis leaflets in fluoroscopy.

Video S7: Fixation of both tricuspid prosthesis leaflets in fluoroscopy.

## Data Availability

The dataset supporting the conclusions of this article is included within the article, and any other inquiry is available from the corresponding author on reasonable request.

## List of abbreviations

COVID-19: coronavirus disease 2019
HES: hypereosinophilic syndrome
hs-CTnI: high-sensitive troponin I
LVEF: left ventricular ejection fraction
MS: mitral stenosis
MR: mitral regurgitation
NT-pro BNP: N terminal pro B type natriuretic peptide
TS: tricuspid stenosis
TR: tricuspid regurgitation
WBC: white blood cell
